# (*E*)-1-[1-(4-Chloro­phen­yl)eth­yl]-3,5-dimethyl-*N*-nitro-1,3,5-triazinan-2-imine

**DOI:** 10.1107/S1600536810046246

**Published:** 2010-11-17

**Authors:** Huai-gang Su, Liang-zhong Xu

**Affiliations:** a College of Chemistry and Molecular Engineering, Qingdao University of Science and Technology, Qingdao 266042, People’s Republic of China

## Abstract

In the title compound, C_13_H_18_ClN_5_O_2_, the 1,3,5-triazinane ring exhibits an envelope conformation with an *E* form. The chloro­phenyl ring and the nitro group are each twisted with respect to the mean plane of the triazinane ring, making dihedral angles of 67.30 (9) and 83.54 (8)°, respectively. In the crystal, weak inter­molecular C—H⋯O hydrogen bonds build up a corrugated layer parallel to the (101) plane.

## Related literature

The title compound was synthesized as a new compound with better insecticidal activity. For similar compounds with insecticidal properties, see: Koln *et al.* (2002 ▶). For related structures, see: Zhao *et al.* (2008 ▶); Hu *et al.* (2008 ▶); Xu *et al.* (2010 ▶) For puckering parameters, see: Cremer & Pople (1975 ▶).
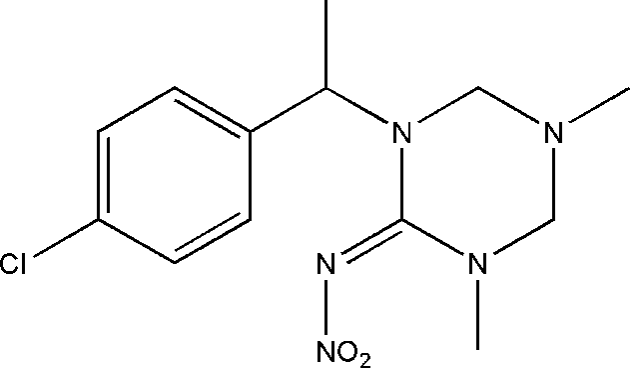


## Experimental

### 

#### Crystal data

C_13_H_18_ClN_5_O_2_
*M* *_r_* = 311.77
Monoclinic, 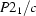

*a* = 7.2483 (14) Å
*b* = 29.568 (6) Å
*c* = 7.2306 (14) Å
β = 108.75 (3)°
*V* = 1467.4 (5) Å^3^
*Z* = 4
Mo *K*α radiation
μ = 0.27 mm^−1^
*T* = 113 K
0.20 × 0.16 × 0.12 mm

#### Data collection

Rigaku Saturn CCD area-detector diffractometer
Absorption correction: multi-scan (*ABSCOR*; Higashi, 1995 ▶) *T*_min_ = 0.947, *T*_max_ = 0.968
9753 measured reflections
2585 independent reflections
2303 reflections with *I* > 2σ(*I*)
*R*_int_ = 0.039

#### Refinement

*R*[*F* ^2^ > 2σ(*F* ^2^)] = 0.041
*wR*(*F* ^2^) = 0.104
*S* = 1.08
2585 reflections
193 parameters
H-atom parameters constrained
Δρ_max_ = 0.46 e Å^−3^
Δρ_min_ = −0.51 e Å^−3^

### 

Data collection: *RAPID-AUTO* (Rigaku, 2004 ▶); cell refinement: *RAPID-AUTO*; data reduction: *RAPID-AUTO*; program(s) used to solve structure: *SHELXTL* (Sheldrick, 2008 ▶); program(s) used to refine structure: *SHELXTL*; molecular graphics: *ORTEPIII* (Burnett & Johnson, 1996 ▶) and *ORTEP-3 for Windows* (Farrugia, 1997 ▶); software used to prepare material for publication: *SHELXTL*.

## Supplementary Material

Crystal structure: contains datablocks I, global. DOI: 10.1107/S1600536810046246/dn2619sup1.cif

Structure factors: contains datablocks I. DOI: 10.1107/S1600536810046246/dn2619Isup2.hkl

Additional supplementary materials:  crystallographic information; 3D view; checkCIF report

## Figures and Tables

**Table 1:** Hydrogen-bond geometry (Å, °)

*D*—H⋯*A*	*D*—H	H⋯*A*	*D*⋯*A*	*D*—H⋯*A*
C8—H8*A*⋯O2^i^	0.98	2.29	3.273 (2)	178
C10—H10*A*⋯O1^ii^	0.99	2.43	3.278 (2)	143
C11—H11*B*⋯O2^i^	0.99	2.49	3.434 (3)	160

Symmetry codes: (i) 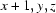; (ii) 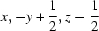.

**Fig. 1.**
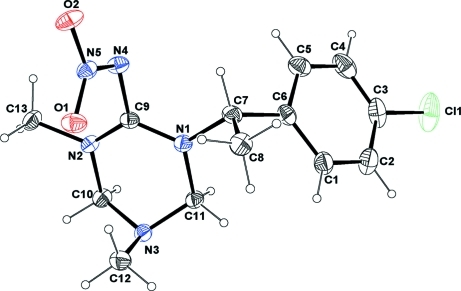
View of the title compound (I) with the atoms labeling scheme. Displacement ellipsoids are drawn at the 50% probability level. H atoms are represented as small spheres of arbitrary radii.

## supplementary crystallographic information

### Comment

The title compound was synthesized as a new compound with better insecticide activity. Lots of similar insecticide compounds with (I) were synthesized (Koln *et al.*, 2002). We report here the crystal struture of (I).

The 1,3,5-triazinan ring exhibits envelope conformation with puckering parameters Q= 0.4777 (19)Å, θ= 57.8 (2)° and φ= 238.5 (3)° (Cremer & Pople, 1975). The chlorophenyl ring as well as the nitro group are twisted with respect to the mean plane of the triazinan ring making dihedral angles of 67.30 (9)° and 83.54 (8)° respectively (Fig. 1). All bond lengths and angles are normal and in a good agreement with those recently reported (Hu *et al.*, 2008; Zhao *et al.*, 2008; Xu *et al.*, 2010).

Weak intermolecular C–H···O hydrogen bonds build up a corrugated layer parallel to the (1 0 1) plane (Table 1).

### Experimental

A mixture of 1,5-dimethyl-2-(nitromethylene)-1,3,5-triazinane (20.64 g, 0.12 mol), potassium carbonate (20.7 g, 0.15 mol), potassium iodization (2 g) and ethyl acetate (150 ml) was stirred and heated in a 500 ml flask. The mixture was slowly heated to 353 K - 363 K and kept for 1 h. Then ,1-chloro-4-(1-chloroethyl)benzene (21 g, 0.12 mol, dissolved in 100 ml of ethyl acetate) was added dropwise into the flask, and the mixture was futher stirred at 353 K - 363 K for 15 h. After cooling, the precipitate was filtered,washed with ethyl acetate and water, and recrystallized from ethyl acetate to obtain flaxen powder. Yield: 86%.

### Refinement

All H atoms were placed in calculated positions, with C–H = 0.93–0.98 Å, and included in the final cycles of refinement using a riding model, with *U*_iso_(H) = 1.2*U*_eq_(C) for aryl, methylene and 1.5*U*_eq_(C) for methyl H atoms.

### Crystal data

**Table:**

C_13_H_18_ClN_5_O_2_	*F*(000) = 656
*M**_r_* = 311.77	*D*_x_ = 1.411 Mg m^−^^3^
Monoclinic, *P*2_1_/*c*	Mo *K*α radiation, λ = 0.71073 Å
Hall symbol: -P 2ybc	Cell parameters from 3278 reflections
*a* = 7.2483 (14) Å	θ = 1.4–27.9°
*b* = 29.568 (6) Å	µ = 0.27 mm^−^^1^
*c* = 7.2306 (14) Å	*T* = 113 K
β = 108.75 (3)°	Needle, colourless
*V* = 1467.4 (5) Å^3^	0.20 × 0.16 × 0.12 mm
*Z* = 4	

### Data collection

**Table:**

Rigaku Saturn CCD area-detector diffractometer	2585 independent reflections
Radiation source: rotating anode	2303 reflections with *I* > 2σ(*I*)
confocal	*R*_int_ = 0.039
Detector resolution: 7.31 pixels mm^-1^	θ_max_ = 25.0°, θ_min_ = 1.4°
ω and φ scans	*h* = −8→8
Absorption correction: multi-scan (*ABSCOR*; Higashi, 1995)	*k* = −35→34
*T*_min_ = 0.947, *T*_max_ = 0.968	*l* = −8→8
9753 measured reflections	

### Refinement

**Table:**

Refinement on *F*^2^	Primary atom site location: structure-invariant direct methods
Least-squares matrix: full	Secondary atom site location: difference Fourier map
*R*[*F*^2^ > 2σ(*F*^2^)] = 0.041	Hydrogen site location: inferred from neighbouring sites
*wR*(*F*^2^) = 0.104	H-atom parameters constrained
*S* = 1.08	*w* = 1/[σ^2^(*F*_o_^2^) + (0.0488*P*)^2^ + 0.7943*P*] where *P* = (*F*_o_^2^ + 2*F*_c_^2^)/3
2585 reflections	(Δ/σ)_max_ < 0.001
193 parameters	Δρ_max_ = 0.46 e Å^−^^3^
0 restraints	Δρ_min_ = −0.51 e Å^−^^3^

### Special details

**Table:**

Geometry. All esds (except the esd in the dihedral angle between two l.s. planes) are estimated using the full covariance matrix. The cell esds are taken into account individually in the estimation of esds in distances, angles and torsion angles; correlations between esds in cell parameters are only used when they are defined by crystal symmetry. An approximate (isotropic) treatment of cell esds is used for estimating esds involving l.s. planes.
Refinement. Refinement of *F*^2^ against ALL reflections. The weighted *R*-factor *wR* and goodness of fit *S* are based on *F*^2^, conventional *R*-factors *R* are based on *F*, with *F* set to zero for negative *F*^2^. The threshold expression of *F*^2^ > σ(*F*^2^) is used only for calculating *R*-factors(gt) *etc*. and is not relevant to the choice of reflections for refinement. *R*-factors based on *F*^2^ are statistically about twice as large as those based on *F*, and *R*- factors based on ALL data will be even larger.

### Fractional atomic coordinates and isotropic or equivalent isotropic displacement parameters (Å^2^)

**Table:**

	*x*	*y*	*z*	*U*_iso_*/*U*_eq_	
Cl1	0.37472 (9)	0.56523 (2)	0.15716 (10)	0.0546 (2)	
O1	−0.25418 (19)	0.29503 (4)	0.41241 (18)	0.0236 (3)	
O2	−0.48276 (18)	0.33395 (5)	0.47562 (18)	0.0254 (3)	
N1	−0.0516 (2)	0.36893 (5)	0.2166 (2)	0.0173 (3)	
N2	−0.2858 (2)	0.32380 (5)	0.0044 (2)	0.0175 (3)	
N3	0.0492 (2)	0.31375 (5)	0.0208 (2)	0.0181 (3)	
N4	−0.3580 (2)	0.36095 (5)	0.2595 (2)	0.0197 (3)	
N5	−0.3641 (2)	0.32910 (5)	0.3838 (2)	0.0181 (3)	
C1	0.3035 (3)	0.44147 (6)	0.3422 (3)	0.0261 (4)	
H1	0.3813	0.4153	0.3853	0.031*	
C2	0.3844 (3)	0.47936 (7)	0.2862 (3)	0.0321 (5)	
H2	0.5159	0.4789	0.2876	0.038*	
C3	0.2725 (3)	0.51775 (7)	0.2283 (3)	0.0323 (5)	
C4	0.0823 (3)	0.51896 (7)	0.2250 (3)	0.0326 (5)	
H4	0.0071	0.5457	0.1871	0.039*	
C5	0.0016 (3)	0.48064 (6)	0.2776 (3)	0.0265 (4)	
H5	−0.1307	0.4813	0.2736	0.032*	
C6	0.1093 (3)	0.44127 (6)	0.3362 (3)	0.0206 (4)	
C7	0.0118 (3)	0.39993 (6)	0.3875 (3)	0.0200 (4)	
H7	−0.1081	0.4105	0.4143	0.024*	
C8	0.1367 (3)	0.37472 (6)	0.5690 (3)	0.0231 (4)	
H8A	0.2520	0.3622	0.5446	0.035*	
H8B	0.0605	0.3501	0.5991	0.035*	
H8C	0.1776	0.3957	0.6797	0.035*	
C9	−0.2273 (2)	0.34984 (5)	0.1609 (2)	0.0166 (4)	
C10	−0.1503 (3)	0.31248 (6)	−0.1043 (3)	0.0199 (4)	
H10A	−0.1808	0.2819	−0.1616	0.024*	
H10B	−0.1683	0.3343	−0.2127	0.024*	
C11	0.0888 (3)	0.35748 (6)	0.1119 (3)	0.0184 (4)	
H11A	0.0810	0.3807	0.0110	0.022*	
H11B	0.2229	0.3578	0.2056	0.022*	
C12	0.0994 (3)	0.27654 (6)	0.1633 (3)	0.0231 (4)	
H12A	0.0757	0.2475	0.0940	0.035*	
H12B	0.0188	0.2785	0.2489	0.035*	
H12C	0.2373	0.2787	0.2420	0.035*	
C13	−0.4771 (3)	0.30192 (6)	−0.0593 (3)	0.0226 (4)	
H13A	−0.5701	0.3204	−0.0193	0.034*	
H13B	−0.4672	0.2719	0.0007	0.034*	
H13C	−0.5222	0.2988	−0.2018	0.034*	

### Atomic displacement parameters (Å^2^)

**Table:**

	*U*^11^	*U*^22^	*U*^33^	*U*^12^	*U*^13^	*U*^23^
Cl1	0.0535 (4)	0.0359 (3)	0.0547 (4)	−0.0206 (3)	−0.0102 (3)	0.0202 (3)
O1	0.0254 (7)	0.0221 (7)	0.0255 (7)	0.0071 (5)	0.0111 (6)	0.0057 (5)
O2	0.0188 (7)	0.0410 (8)	0.0203 (7)	0.0038 (6)	0.0120 (5)	0.0028 (6)
N1	0.0174 (8)	0.0186 (7)	0.0171 (7)	0.0002 (6)	0.0071 (6)	−0.0011 (6)
N2	0.0142 (7)	0.0210 (8)	0.0176 (7)	0.0000 (6)	0.0057 (6)	0.0004 (6)
N3	0.0165 (8)	0.0198 (8)	0.0185 (7)	−0.0008 (6)	0.0062 (6)	−0.0019 (6)
N4	0.0190 (8)	0.0220 (8)	0.0207 (8)	0.0039 (6)	0.0100 (6)	0.0035 (6)
N5	0.0151 (7)	0.0233 (8)	0.0155 (7)	0.0002 (6)	0.0045 (6)	−0.0007 (6)
C1	0.0241 (10)	0.0198 (9)	0.0300 (10)	−0.0014 (7)	0.0026 (8)	0.0005 (8)
C2	0.0260 (11)	0.0310 (11)	0.0346 (11)	−0.0077 (8)	0.0033 (9)	0.0021 (9)
C3	0.0390 (12)	0.0229 (10)	0.0254 (10)	−0.0100 (9)	−0.0032 (9)	0.0030 (8)
C4	0.0439 (13)	0.0204 (10)	0.0262 (11)	0.0063 (9)	0.0012 (9)	0.0000 (8)
C5	0.0286 (10)	0.0269 (10)	0.0223 (10)	0.0049 (8)	0.0059 (8)	−0.0039 (8)
C6	0.0249 (10)	0.0188 (9)	0.0164 (9)	−0.0012 (7)	0.0044 (7)	−0.0038 (7)
C7	0.0206 (9)	0.0214 (9)	0.0184 (9)	0.0015 (7)	0.0067 (7)	−0.0046 (7)
C8	0.0247 (10)	0.0262 (10)	0.0189 (9)	−0.0005 (8)	0.0075 (8)	−0.0005 (7)
C9	0.0175 (9)	0.0155 (8)	0.0165 (8)	0.0039 (7)	0.0052 (7)	0.0038 (7)
C10	0.0196 (9)	0.0253 (10)	0.0159 (8)	−0.0008 (7)	0.0073 (7)	−0.0021 (7)
C11	0.0175 (9)	0.0204 (9)	0.0195 (9)	−0.0012 (7)	0.0088 (7)	−0.0010 (7)
C12	0.0223 (10)	0.0215 (9)	0.0250 (10)	0.0027 (7)	0.0070 (8)	0.0004 (7)
C13	0.0146 (9)	0.0290 (10)	0.0232 (9)	−0.0024 (7)	0.0045 (7)	−0.0022 (8)

### Geometric parameters (Å, °)

**Table:**

Cl1—C3	1.740 (2)	C4—C5	1.383 (3)
O1—N5	1.2595 (19)	C4—H4	0.9500
O2—N5	1.2522 (19)	C5—C6	1.390 (3)
N1—C9	1.332 (2)	C5—H5	0.9500
N1—C7	1.488 (2)	C6—C7	1.516 (3)
N1—C11	1.490 (2)	C7—C8	1.527 (3)
N2—C9	1.321 (2)	C7—H7	1.0000
N2—C13	1.464 (2)	C8—H8A	0.9800
N2—C10	1.480 (2)	C8—H8B	0.9800
N3—C11	1.437 (2)	C8—H8C	0.9800
N3—C10	1.439 (2)	C10—H10A	0.9900
N3—C12	1.471 (2)	C10—H10B	0.9900
N4—N5	1.312 (2)	C11—H11A	0.9900
N4—C9	1.395 (2)	C11—H11B	0.9900
C1—C2	1.384 (3)	C12—H12A	0.9800
C1—C6	1.394 (3)	C12—H12B	0.9800
C1—H1	0.9500	C12—H12C	0.9800
C2—C3	1.379 (3)	C13—H13A	0.9800
C2—H2	0.9500	C13—H13B	0.9800
C3—C4	1.372 (3)	C13—H13C	0.9800
			
C9—N1—C7	121.49 (14)	C6—C7—H7	107.3
C9—N1—C11	119.58 (14)	C8—C7—H7	107.3
C7—N1—C11	118.87 (13)	C7—C8—H8A	109.5
C9—N2—C13	122.48 (15)	C7—C8—H8B	109.5
C9—N2—C10	120.15 (14)	H8A—C8—H8B	109.5
C13—N2—C10	117.16 (14)	C7—C8—H8C	109.5
C11—N3—C10	108.91 (14)	H8A—C8—H8C	109.5
C11—N3—C12	112.59 (14)	H8B—C8—H8C	109.5
C10—N3—C12	113.18 (14)	N2—C9—N1	121.19 (15)
N5—N4—C9	111.03 (14)	N2—C9—N4	119.45 (15)
O2—N5—O1	120.71 (14)	N1—C9—N4	119.10 (15)
O2—N5—N4	117.46 (14)	N3—C10—N2	111.35 (14)
O1—N5—N4	121.83 (14)	N3—C10—H10A	109.4
C2—C1—C6	120.84 (18)	N2—C10—H10A	109.4
C2—C1—H1	119.6	N3—C10—H10B	109.4
C6—C1—H1	119.6	N2—C10—H10B	109.4
C3—C2—C1	119.5 (2)	H10A—C10—H10B	108.0
C3—C2—H2	120.3	N3—C11—N1	111.63 (14)
C1—C2—H2	120.3	N3—C11—H11A	109.3
C4—C3—C2	121.07 (19)	N1—C11—H11A	109.3
C4—C3—Cl1	119.65 (16)	N3—C11—H11B	109.3
C2—C3—Cl1	119.28 (18)	N1—C11—H11B	109.3
C3—C4—C5	119.04 (18)	H11A—C11—H11B	108.0
C3—C4—H4	120.5	N3—C12—H12A	109.5
C5—C4—H4	120.5	N3—C12—H12B	109.5
C4—C5—C6	121.61 (19)	H12A—C12—H12B	109.5
C4—C5—H5	119.2	N3—C12—H12C	109.5
C6—C5—H5	119.2	H12A—C12—H12C	109.5
C5—C6—C1	117.94 (17)	H12B—C12—H12C	109.5
C5—C6—C7	119.32 (17)	N2—C13—H13A	109.5
C1—C6—C7	122.73 (16)	N2—C13—H13B	109.5
N1—C7—C6	109.73 (14)	H13A—C13—H13B	109.5
N1—C7—C8	110.65 (14)	N2—C13—H13C	109.5
C6—C7—C8	114.18 (15)	H13A—C13—H13C	109.5
N1—C7—H7	107.3	H13B—C13—H13C	109.5

### Hydrogen-bond geometry (Å, °)

**Table:**

*D*—H···*A*	*D*—H	H···*A*	*D*···*A*	*D*—H···*A*
C8—H8A···O2^i^	0.98	2.29	3.273 (2)	178
C10—H10A···O1^ii^	0.99	2.43	3.278 (2)	143
C11—H11B···O2^i^	0.99	2.49	3.434 (3)	160

Symmetry codes: (i) *x*+1, *y*, *z*; (ii) *x*, −*y*+1/2, *z*−1/2.
